# Dynamic monitoring of immunoglobulin G as a prognostic indicator after curative resection in high-risk stage II–III colorectal cancer: a retrospective cohort study

**DOI:** 10.3389/fonc.2026.1753074

**Published:** 2026-03-04

**Authors:** Junyi Sun, Feng Guo, Yinfang Guo, Ni Wang, Jiangxue Feng

**Affiliations:** 1 Medical Laboratory Center, Hebei Hospital of Traditional Chinese Medicine, Shijiazhuang, Hebei, China; 2 Nursing Profession, Hebei University of Chinese Medicine, Shijiazhuang, Hebei, China; 3 Aonrectal Department 1, Hebei Hospital of Traditional Chinese Medicine, Shijiazhuang, Hebei, China; 4 Gastrointestinal Oncology Surgery, Hebei Hospital of Traditional Chinese Medicine, Shijiazhuang, Hebei, China

**Keywords:** colorectal cancer, disease-free survival, dynamic monitoring, immunoglobulin G, prognostic biomarkers

## Abstract

**Background:**

Reliable markers for predicting postoperative recurrence in high-risk stage II–III colorectal cancer remain limited. Dynamic changes in immunoglobulin G (IgG) may provide prognostic information beyond carcinoembryonic antigen (CEA).

**Methods:**

This single-centre retrospective cohort study included 192 patients with high-risk stage II or III colorectal cancer who underwent curative resection between January 2021 and June 2023. The study evaluated whether dynamic postoperative monitoring of serum IgG predicts 2-year disease-free survival (DFS) compared with CEA. Serial IgG and CEA measurements within 24 months were categorised as favourable or unfavourable trajectories based on predefined thresholds and temporal trends. Patients were further stratified into four groups according to combined IgG and CEA dynamics. Survival was assessed using Kaplan–Meier analysis and Cox regression models.

**Results:**

Among 192 eligible patients, 60 (31.3%) experienced recurrence or death within 2 years. Unfavourable IgG trajectories (n=82) were associated with significantly lower 2-year DFS than favourable trajectories (55% [95% CI 44–65] vs 82% [95% CI 73–88], log-rank P<0.01). CEA trajectories showed only borderline separation (67% [95% CI 55–77] vs 79% [95% CI 71–85], log-rank P = 0.06). Patients with both unfavourable IgG and CEA trajectories had the poorest outcomes (2-year DFS 31% [95% CI 16–47]). In multivariable analysis, an unfavourable IgG trajectory independently predicted recurrence or death (HR 2.05, 95% CI 1.32–3.18), whereas CEA trajectory was not significant.

**Conclusion:**

Dynamic postoperative IgG monitoring is independently associated with disease recurrence in high-risk stage II–III colorectal cancer and offers incremental prognostic value beyond CEA. Incorporating serial IgG measurements may enhance postoperative risk stratification, although confirmation in prospective multicentre studies is warranted.

## 1 Introduction

Colorectal cancer remains one of the leading causes of cancer-related morbidity and mortality worldwide, and despite advances in surgical techniques and systemic therapies, recurrence after curative resection continues to pose a major clinical challenge (1, 2). Patients with high-risk stage II and stage III disease represent a particularly vulnerable subgroup, for whom accurate prognostic assessment is essential to guide adjuvant treatment strategies and optimise surveillance schedules (3). Although the TNM staging system forms the cornerstone of risk stratification, outcomes vary substantially even among patients with the same pathological stage, underscoring the need for more reliable biomarkers to refine prognostic prediction (4, 5).

Serological markers such as carcinoembryonic antigen (CEA) are widely used in routine practice for monitoring disease recurrence, yet their sensitivity and specificity remain suboptimal (2, 6). Similarly, pathological features and molecular alterations, including KRAS mutation, lymphovascular invasion, and microsatellite instability, provide additional information but fail to fully capture the complex interplay between host biology and tumour progression (7–9). In this context, attention has increasingly turned toward immune-related parameters, which may reflect systemic responses to malignancy and thus hold promise as complementary prognostic indicators.

Immunoglobulin G (IgG), as the predominant circulating immunoglobulin, plays a critical role in humoral immunity and tumour immune surveillance (10, 11). Prior studies have suggested associations between altered IgG levels and cancer risk or progression; however, evidence in colorectal cancer is sparse, and most available analyses have focused on single preoperative measurements (1, 12, 13). The potential prognostic value of dynamic IgG monitoring, reflecting longitudinal immune status over the postoperative period, remains largely unexplored (14, 15). Considering the recognised limitations of CEA and other conventional biomarkers, exploring whether immunoglobulin trajectories provide incremental prognostic information is of considerable clinical interest.

The present study was therefore undertaken to evaluate the prognostic significance of serial IgG monitoring in patients with high-risk stage II and stage III colorectal cancer following curative resection. By comparing the predictive performance of IgG trajectories with that of CEA, and by examining their combined utility, this study aims to clarify the potential role of immunoglobulin dynamics in refining risk stratification. Such insights may help identify patients at higher risk of recurrence who could benefit from more intensive surveillance or tailored therapeutic strategies.

## 2 Methods

### 2.1 Study design and patient population

This study was designed as a retrospective cohort analysis conducted at a tertiary referral centre with a dedicated colorectal cancer unit. Consecutive patients undergoing curative-intent surgical resection for colorectal adenocarcinoma between January 2021 and June 2023 were screened. Eligibility was limited to individuals with high-risk stage II or stage III disease, defined in accordance with the American Joint Committee on Cancer 8th edition staging system. Patients were required to have complete clinicopathological information, baseline and follow-up immunoglobulin measurements, and systematic surveillance data.

Exclusion criteria included the presence of concomitant autoimmune or haematological disorders that could affect immunoglobulin concentrations, prior exposure to immunomodulatory or immunosuppressive agents, receipt of any neoadjuvant (preoperative) chemotherapy and/or radiotherapy, and incomplete or missing survival or laboratory data. From the 384 patients initially reviewed, 192 fulfilled all requirements and formed the final analytic cohort. This rigorous selection process was intended to ensure homogeneity of the study population and to reduce potential biases from heterogeneous disease characteristics, treatment exposure, or incomplete datasets.

### 2.2 Clinical and pathological assessment

Demographic information, comorbidities, and perioperative clinical details were abstracted from the institutional electronic medical records. Staging was performed according to contemporary oncological standards, with imaging work-up complemented by histopathological verification. High-risk features in stage II patients were defined as T4 disease, examination of fewer than 12 lymph nodes, poorly differentiated histology, or evidence of lymphovascular or perineural invasion. Pathology slides were re-examined by two senior gastrointestinal pathologists blinded to clinical outcomes to ensure diagnostic consistency.

Histological parameters recorded included tumour site, size, grade of differentiation, and presence of lymphovascular or perineural invasion. Molecular profiling comprised KRAS, NRAS, and BRAF mutational analysis via polymerase chain reaction or targeted next-generation sequencing, while microsatellite instability and mismatch repair status were determined by immunohistochemistry. TP53 mutations were noted when available. These molecular factors were incorporated due to their recognised prognostic implications and their integration into routine clinical decision-making.

### 2.3 Laboratory measurements

Serum immunoglobulin concentrations (IgG, IgA, and IgM) were quantified using an immunonephelometric assay (BECKMAN COULTER, IMMAGE 800) performed in the hospital’s central laboratory. CEA and LDH levels were determined through electrochemiluminescence immunoassays (BECKMAN COULTER, AU5800). Blood samples were collected in the fasting state within one week prior to surgery, and subsequent measurements were obtained during postoperative surveillance visits at 3- to 6-month intervals. For analytic purposes, postoperative measurements were aligned to nominal 6-, 12-, 18-, and 24-month time points using the closest available value within the corresponding surveillance window; when recurrence/death occurred before the first 6-month assessment, the available earlier postoperative measurement(s) obtained during routine follow-up were used for trajectory assignment. All assays were conducted in compliance with manufacturer recommendations and subjected to internal quality control.

Dynamic monitoring was achieved by serially tracking immunoglobulin and tumour marker values across the postoperative period. For analytic purposes, trajectories were categorised as “favourable” when values remained stable or declined within the physiological range, and as “unfavourable” when progressive elevation or persistently high levels were observed. Laboratory personnel were blinded to patient clinical outcomes during testing, thereby reducing the risk of measurement bias.

### 2.4 Treatment and follow-up

Surgical procedures were performed according to international oncological guidelines, with either laparoscopic or open approaches determined by tumour characteristics and surgeon expertise. Radical resection with negative margins was achieved in all patients. Postoperative adjuvant chemotherapy was recommended for all stage III and selected high-risk stage II cases, typically consisting of oxaliplatin-based doublets (FOLFOX or CAPOX) for a duration of six months. Compliance with chemotherapy was assessed by the proportion of patients completing ≥80% of prescribed cycles.

Patients were followed prospectively through outpatient visits every three months during the first two years and biannually thereafter. Surveillance included clinical examination, serum tumour markers (CEA, immunoglobulins, LDH), colonoscopy, and cross-sectional imaging as indicated. The primary study endpoint was 2-year DFS, defined as the time from surgery to the first documented recurrence or death from any cause. Patients without events were censored at the date of last follow-up.

### 2.5 Definitions of dynamic trajectories

Dynamic trajectories of IgG and CEA were assessed using serial postoperative measurements obtained during routine surveillance. For classification, the baseline postoperative value was defined as the earliest available postoperative measurement after surgery (typically at the first follow-up visit within 3–6 months and prior to any documented DFS event), and percentage changes were calculated relative to this baseline. For IgG, a favourable trajectory was defined as: (1) IgG remaining within the institutional reference range (7–16 g/L) with subsequent variation within ±15% of the baseline postoperative value, or (2) a ≥15% reduction from the baseline postoperative value that was maintained (≤15% variation) across at least two consecutive follow-up points. An unfavourable IgG trajectory was defined as either: (1) persistent elevation of IgG above the upper limit of normal (≥16 g/L) observed at least twice consecutively, or (2) a sustained rise of ≥15% from the baseline postoperative value at two or more consecutive visits, regardless of absolute level. This dual criterion was designed to capture both absolute hypergammaglobulinaemia and abnormal upward trends within the normal range. The 15% threshold was prespecified to exceed expected analytical and short-term biological variability of immunonephelometric IgG measurements and to represent a potentially clinically meaningful change; however, sensitivity analyses across alternative cut-offs were not performed in this exploratory cohort, and the optimal threshold requires external validation.

CEA trajectories were defined analogously using a reference cut-off of 5 ng/mL. A favourable CEA trajectory was defined as persistently ≤5 ng/mL, or a >50% reduction from the baseline postoperative value with subsequent values remaining ≤5 ng/mL. An unfavourable CEA trajectory was defined as persistent elevation >5 ng/mL observed at least twice consecutively, or a sustained rise ≥25% from the individual’s postoperative nadir across two or more consecutive visits.

**Figure 1:**
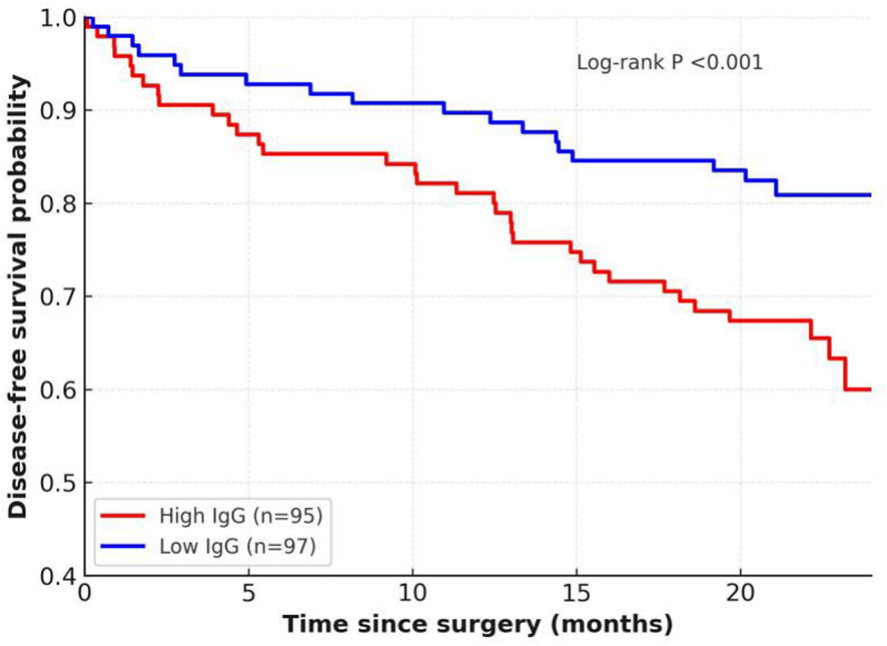
Kaplan–Meier curves of 2-year DFS according to preoperative serum IgG levels (high vs low, dichotomised at the cohort median). Number at risk is shown below the x-axis.

For exploratory purposes, patients were further stratified into four composite groups based on combined IgG and CEA dynamics: both favourable, IgG favourable/CEA unfavourable, IgG unfavourable/CEA favourable, and both unfavourable. These trajectory definitions were prespecified to reduce *post hoc* bias and enhance reproducibility in future studies. In addition, for descriptive baseline analysis, preoperative IgG was dichotomised at the cohort median (high vs low) for Kaplan–Meier analysis (**Figure 1**).

### 2.6 Ethics

The current study was approved by the Ethics Committee of the Hebei Hospital of Traditional Chinese Medicine (approval number HTH202508202). Given its retrospective nature, the requirement for individual written informed consent was waived, although data confidentiality and anonymity were strictly maintained. All procedures were conducted in accordance with the principles of the Declaration of Helsinki and relevant national regulations governing human subject research. Data were stored securely within the institutional research database with access restricted to study investigators.

### 2.7 Statistical analysis

Descriptive statistics were used to summarise baseline characteristics, expressed as mean ± standard deviation for continuous variables and as frequencies with percentages for categorical variables. Group comparisons were performed using Student’s t-test or the Mann–Whitney U test for continuous data, and χ² or Fisher’s exact test for categorical data, as appropriate.

Disease-free survival was estimated using the Kaplan–Meier method, and differences between groups were compared by log-rank tests; 2-year DFS estimates are reported with 95% confidence intervals (CI). Univariate Cox proportional hazards regression was performed to identify potential prognostic factors among variables with adequate completeness, followed by multivariable Cox regression incorporating variables with P<0.10 on univariate analysis and those of clinical relevance. Hazard ratios (HR) with 95% CI were calculated. Because biomarker trajectories are derived from serial measurements accrued during follow-up, time-dependent covariate modelling or landmark analysis would be preferable; however, in this exploratory retrospective study we summarised trajectories as categorical variables and entered them as fixed covariates, and the potential for guarantee-time bias is discussed as a limitation. Statistical significance was defined as a two-sided P value <0.05. Analyses were conducted using SPSS version 27.0 (IBM Corp.) and R version 4.2.

## 3 Results

### 3.1 Baseline characteristics of the study cohort

**Figure 2:**
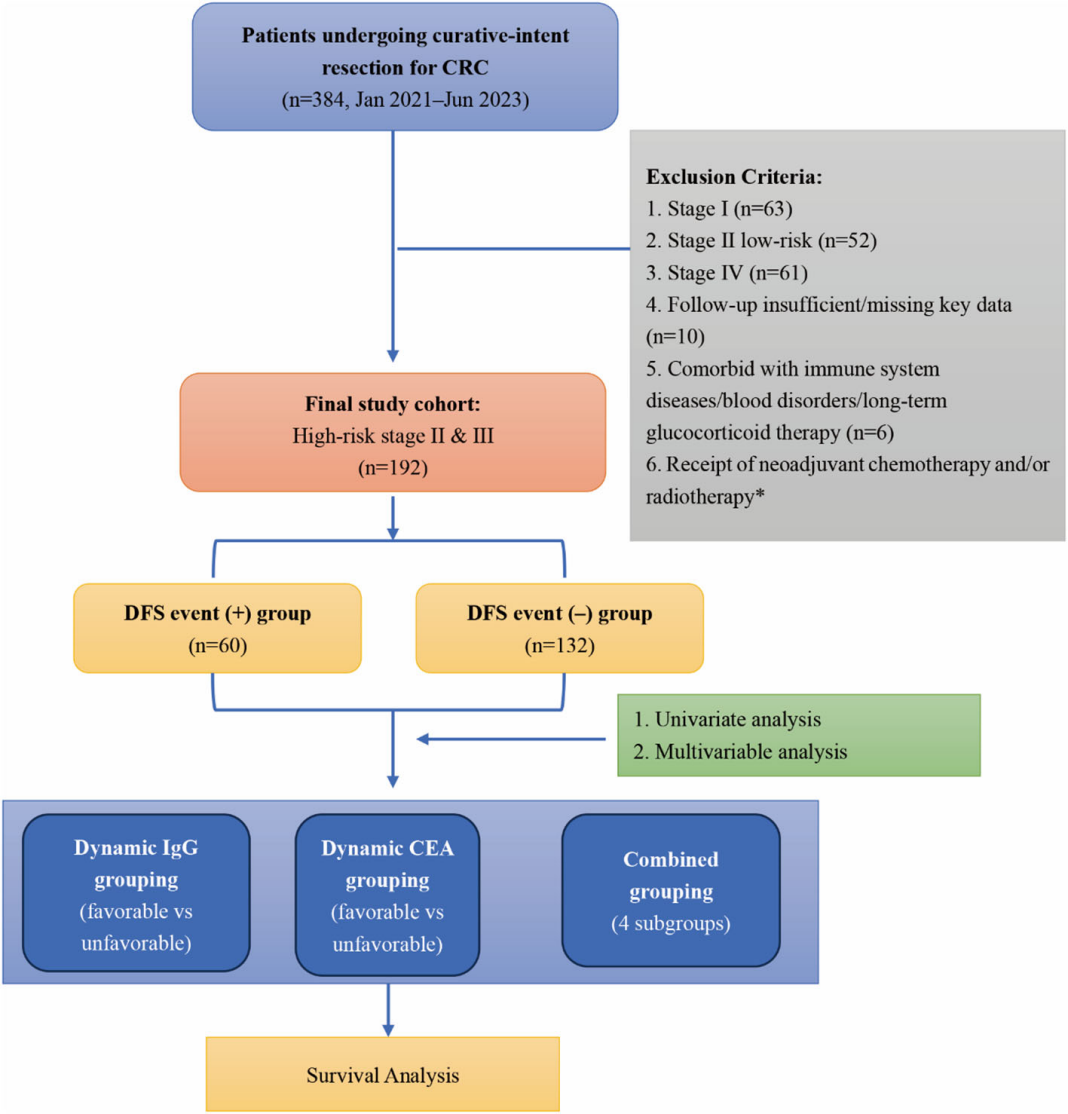
Flow diagram of patient selection and study cohort. *Patients receiving neoadjuvant therapy were excluded during screening.

A total of 384 patients who underwent curative-intent resection for colorectal cancer between January 2021 and June 2023 were screened, of whom 192 patients with high-risk stage II or stage III disease fulfilled the eligibility criteria and were included in the analysis. Based on 2-year DFS, patients were stratified into those with DFS events (n=60, 31.3%) and those without events (n=132, 68.7%). Baseline demographics and comorbidities are summarised in **Table 1**. The mean age was slightly higher in the event group compared with the non-event group (63 ± 8 vs 61 ± 9 years, P = 0.14). The proportion of male patients was similar between groups (60.0% [36/60] vs 56.1% [74/132], P = 0.63). The prevalence of hypertension (40.0% [24/60] vs 32.6% [43/132], P = 0.31), diabetes (23.3% [14/60] vs 15.9% [21/132], P = 0.24), and coronary artery disease (13.3% [8/60] vs 7.6% [10/132], P = 0.24) did not differ significantly. Baseline renal and hepatic function were comparable, with mean serum creatinine of 78 ± 13 µmol/L in the event group versus 76 ± 12 µmol/L in the non-event group (P = 0.56). Mean albumin was lower in the event group (39.5 ± 4.2 g/L vs 41.0 ± 4.5 g/L, P = 0.08), with corresponding differences in the prognostic nutritional index (47.2 ± 5.8 vs 48.8 ± 6.1, P = 0.07). Among serological markers, elevated CEA was more frequent in the event group (38.3% [23/60] vs 24.2% [32/132], P = 0.05), and LDH elevation was also more common (15.0% [9/60] vs 6.1% [8/132], P = 0.05). Preoperative IgG levels were significantly higher in the event group compared with the non-event group (14.2 ± 2.5 vs 12.7 ± 2.3 g/L, P<0.01), whereas IgA and IgM concentrations showed no statistically significant differences between groups, although IgA levels demonstrated a trend toward lower values in patients with DFS events (**Table 1**). The patient selection flow diagram is shown in **Figure 2**.

**Table 1:** Demographics, comorbidities, functional and serological characteristics according to 2-year DFS status (n=192).

Characteristic	DFS event (+) (n=60)	DFS event (–) (n=132)	Statistical test (value)	P value
Demographics				
Age, years, mean ± SD	63 ± 8	61 ± 9	t-test (t=1.47)	0.14
Male sex, n (%)	60.0% (36/60)	56.1% (74/132)	χ²-test (χ²=0.23)	0.63
BMI, kg/m², mean ± SD	24.1 ± 3.2	23.7 ± 3.1	t-test (t=0.80)	0.42
ECOG PS ≥2, n (%)	6.7% (4/60)	3.8% (5/132)	χ²-test (χ²=0.52)	0.47
Comorbidities				
Hypertension, n (%)	40.0% (24/60)	32.6% (43/132)	χ²-test (χ²=1.04)	0.31
Diabetes, n (%)	23.3% (14/60)	15.9% (21/132)	χ²-test (χ²=1.36)	0.24
Coronary artery disease, n (%)	13.3% (8/60)	7.6% (10/132)	χ²-test (χ²=1.39)	0.24
Chronic kidney disease (eGFR <60), n (%)	10.0% (6/60)	4.5% (6/132)	χ²-test (χ²=1.95)	0.16
Chronic lung disease, n (%)	10.0% (6/60)	6.8% (9/132)	χ²-test (χ²=0.46)	0.50
Functional parameters				
Systolic BP, mmHg, mean ± SD	131 ± 12	129 ± 11	t-test (t=0.69)	0.49
Diastolic BP, mmHg, mean ± SD	81 ± 8	80 ± 7	t-test (t=0.78)	0.44
Serum creatinine, µmol/L, mean ± SD	78 ± 13	76 ± 12	t-test (t=0.58)	0.56
ALT, U/L, mean ± SD	28 ± 9	27 ± 8	t-test (t=0.72)	0.47
AST, U/L, mean ± SD	27 ± 8	26 ± 7	t-test (t=0.81)	0.42
Albumin, g/L, mean ± SD	39.5 ± 4.2	41.0 ± 4.5	t-test (t=1.76)	0.08
PNI, mean ± SD	47.2 ± 5.8	48.8 ± 6.1	t-test (t=1.82)	0.07
Serological markers				
CEA >5 ng/mL, n (%)	38.3% (23/60)	24.2% (32/132)	χ²-test (χ²=3.87)	0.05*
CA19–9 elevated, n (%)	25.0% (15/60)	18.2% (24/132)	χ²-test (χ²=1.13)	0.29
NLR ≥3, n (%)	46.7% (28/60)	34.1% (45/132)	χ²-test (χ²=2.58)	0.11
PLR ≥150, n (%)	43.3% (26/60)	33.3% (44/132)	χ²-test (χ²=1.63)	0.20
SII, mean ± SD	560 ± 180	505 ± 170	t-test (t=1.56)	0.12
LDH elevated, n (%)	15.0% (9/60)	6.1% (8/132)	χ²-test (χ²=3.87)	0.05*
Immunoglobulins (preoperative)				
IgG, g/L, mean ± SD	14.2 ± 2.5	12.7 ± 2.3	t-test (t=3.63)	<0.01*
IgA, g/L, mean ± SD	2.9 ± 0.8	2.6 ± 0.7	t-test (t=1.75)	0.08
IgM, g/L, mean ± SD	1.1 ± 0.4	1.0 ± 0.3	t-test (t=1.59)	0.12

Values are presented as mean ± SD for continuous variables and as % (n/N) for categorical variables, unless otherwise specified. P values were derived from Student’s t test for continuous variables and χ² test (or Fisher’s exact test when appropriate) for categorical variables. *P<0.05 significant.

DFS, disease-free survival; ECOG PS, Eastern Cooperative Oncology Group performance status; BMI, body mass index; eGFR, estimated glomerular filtration rate; ALT, alanine aminotransferase; AST, aspartate aminotransferase; PNI, prognostic nutritional index; NLR, neutrophil-to-lymphocyte ratio; PLR, platelet-to-lymphocyte ratio; SII, systemic immune-inflammation index; LDH, lactate dehydrogenase; CEA, carcinoembryonic antigen; CA19-9, carbohydrate antigen 19-9; Ig, immunoglobulin.

Tumour-related characteristics are detailed in **Table 2**. The distribution of primary site was balanced between groups (right colon 35.0% [21/60] vs 29.5% [39/132]; left colon 40.0% [24/60] vs 43.9% [58/132]; rectum 25.0% [15/60] vs 26.5% [35/132]; P = 0.71). A higher proportion of stage III disease was observed among patients with DFS events (76.7% [46/60]) compared with those without events (67.4% [89/132], P = 0.32). Pathological features including poor differentiation (18.3% [11/60] vs 13.6% [18/132], P = 0.40), lymphovascular invasion (36.7% [22/60] vs 27.3% [36/132], P = 0.20), and perineural invasion (26.7% [16/60] vs 19.7% [26/132], P = 0.30) were not significantly different. Among molecular markers, KRAS mutation was more common in the event group (53.8% [28/52] vs 34.7% [41/118], P = 0.02), whereas NRAS, BRAF, MSI-H/dMMR, and p53 mutation showed no significant differences. Treatment patterns were comparable, with laparoscopic surgery performed in 61.7% (37/60) and 68.2% (90/132) of patients (P = 0.39), and adjuvant chemotherapy administered in 76.7% (46/60) versus 75.0% (99/132) (P = 0.80). Completion of ≥80% of planned chemotherapy cycles was slightly lower in the event group (76.1% [35/46] vs 85.9% [85/99], P = 0.17) (**Table 2**).

**Table 2:** Tumour-related and treatment characteristics according to 2-year DFS status (n=192).

Characteristic	DFS event (+) (n=60)	DFS event (–) (n=132)	Statistical test (value)	P value
Tumour site			χ²-test (χ²=0.68)	0.71
Right colon, n (%)	35.0% (21/60)	29.5% (39/132)		
Left colon, n (%)	40.0% (24/60)	43.9% (58/132)		
Rectum, n (%)	25.0% (15/60)	26.5% (35/132)		
Stage			χ²-test (χ²=0.97)	0.32
High-risk stage II, n (%)	23.3% (14/60)	32.6% (43/132)		
Stage III, n (%)	76.7% (46/60)	67.4% (89/132)		
Tumour size, cm, mean ± SD	5.1 ± 1.2	4.8 ± 1.1	t-test (t=1.09)	0.28
Poor differentiation/other histology, n (%)	18.3% (11/60)	13.6% (18/132)	χ²-test (χ²=0.71)	0.40
Lymphovascular invasion, n (%)	36.7% (22/60)	27.3% (36/132)	χ²-test (χ²=1.63)	0.20
Perineural invasion, n (%)	26.7% (16/60)	19.7% (26/132)	χ²-test (χ²=1.05)	0.30
Lymph nodes retrieved ≥12, n (%)	85.0% (51/60)	88.6% (117/132)	χ²-test (χ²=0.52)	0.47
Molecular profile**^#^**				
KRAS mutation, n (%)	53.8% (28/52)	34.7% (41/118)	χ²-test (χ²=5.24)	0.02*
NRAS mutation, n (%)	6.3% (3/48)	3.5% (4/114)	χ²-test (χ²=0.59)	0.44
BRAF V600E mutation, n (%)	10.0% (5/50)	6.0% (7/117)	χ²-test (χ²=0.80)	0.37
MSI-H/dMMR, n (%)	5.2% (3/58)	9.3% (12/129)	χ²-test (χ²=0.95)	0.33
p53 mutation, n (%)	50.0% (25/50)	53.0% (61/115)	χ²-test (χ²=0.13)	0.72
Treatment				
Laparoscopic surgery, n (%)	61.7% (37/60)	68.2% (90/132)	χ²-test (χ²=0.74)	0.39
Adjuvant chemotherapy, n (%)	76.7% (46/60)	75.0% (99/132)	χ²-test (χ²=0.06)	0.80
– FOLFOX	33.3% (20/60)	32.6% (43/132)		
– CAPOX	31.7% (19/60)	29.5% (39/132)		
– Capecitabine/5-FU monotherapy	11.7% (7/60)	12.9% (17/132)		
Chemotherapy completion ≥80%, n (%)	76.1% (35/46)	85.9% (85/99)	χ²-test (χ²=1.86)	0.17

Values are presented as mean ± SD for continuous variables and as % (n/N) for categorical variables, unless otherwise specified. P values were derived from Student’s t test for continuous variables and χ² test (or Fisher’s exact test when appropriate) for categorical variables. **^#^**Molecular testing was not available for all patients; denominators indicate the number of patients tested. *P<0.05 significant.

### 3.2 Univariate analysis of prognostic factors

Univariate Cox proportional hazards analysis was performed to evaluate the association of baseline clinical, pathological, and serological factors with 2-year DFS. As shown in **Figure 1**, patients with high preoperative IgG (high vs low, dichotomised at the cohort median; n=95 vs n=97) had significantly lower DFS than those with low preoperative IgG, with estimated 2-year DFS of 60% (95% CI 49–69) versus 81% (95% CI 72–88) (log-rank P<0.001).

The results of the univariate Cox regression are summarised in **Table 3**. Higher preoperative IgG was significantly associated with increased risk of recurrence or death (HR 1.18 per 1 g/L increase, 95% CI 1.08–1.28, P<0.01). In addition, KRAS mutation (HR 1.85, 95% CI 1.11–3.08, P = 0.02), elevated CEA (>5 ng/mL; HR 1.62, 95% CI 1.00–2.63, P = 0.05), and elevated LDH (HR 1.84, 95% CI 1.02–3.32, P = 0.04) were also associated with adverse DFS. Other demographic factors (e.g., age, sex, BMI, comorbidities), pathological features (tumour size, stage, histology, lymphovascular or perineural invasion), and treatment-related variables (surgical approach, chemotherapy administration, and completion) were not significantly associated with DFS (all P>0.10).

**Table 3:** Univariate Cox regression analysis (selected variables with adequate completeness).

Variable	HR	95% CI	Statistical test (Wald χ²)	P value
Age, years	1.02	0.99–1.05	χ²=2.16	0.14
Sex (male vs female)	1.08	0.67–1.74	χ²=0.11	0.74
BMI, kg/m²	0.97	0.91–1.03	χ²=0.98	0.32
ECOG PS ≥2 (vs 0–1)	1.45	0.55–3.85	χ²=0.55	0.46
Hypertension	1.18	0.71–1.95	χ²=0.41	0.52
Diabetes	1.33	0.71–2.50	χ²=0.78	0.38
Albumin, g/L	0.95	0.89–1.01	χ²=3.05	0.08
Prognostic nutritional index (continuous)	0.97	0.93–1.01	χ²=2.92	0.09
CEA >5 ng/mL	1.62	1.00–2.63	χ²=3.84	0.05*
LDH elevated	1.84	1.02–3.32	χ²=4.10	0.04*
IgG (per 1 g/L increase)	1.18	1.08–1.28	χ²=12.93	<0.01*
KRAS mutation	1.85	1.11–3.08	χ²=5.35	0.02*
Tumour stage (III vs II-high risk)	1.42	0.80–2.51	χ²=1.42	0.23
Tumour size, cm	1.11	0.96–1.28	χ²=1.98	0.16
Poor differentiation	1.31	0.67–2.55	χ²=0.63	0.43
Lymphovascular invasion (yes vs no)	1.47	0.87–2.48	χ²=2.06	0.15
Perineural invasion (yes vs no)	1.38	0.78–2.44	χ²=1.26	0.26

Values are HR with 95% CI from univariate Cox proportional hazards regression. ECOG PS, Eastern Cooperative Oncology Group performance status. *P<0.05 significant.

Collectively, these findings indicate that both established markers (CEA, KRAS) and metabolic/immune parameters (LDH, IgG) were associated with DFS in univariate analysis, with IgG emerging as a robust risk factor warranting further multivariate evaluation.

### 3.3 Multivariable analysis of prognostic factors

In the multivariable Cox regression analysis including IgG, KRAS mutation, CEA, LDH, albumin, and stage, preoperative serum IgG and KRAS mutation remained independent predictors of 2-year DFS (**Table 4**). Elevated IgG was associated with a higher risk of recurrence or death (HR 1.16 per 1 g/L increase, 95% CI 1.06–1.27, P<0.01), and KRAS mutation was also independently associated with adverse DFS (HR 1.78, 95% CI 1.05–3.01, P = 0.03).

**Table 4:** Multivariable Cox regression analysis of prognostic factors.

Variable	HR	95% CI	Statistical test (Wald χ²)	P value
IgG (per 1 g/L increase)	1.16	1.06–1.27	χ²=9.62	<0.01*
KRAS mutation	1.78	1.05–3.01	χ²=4.62	0.03*
CEA >5 ng/mL	1.52	0.95–2.46	χ²=3.02	0.08
LDH elevated	1.69	0.94–3.06	χ²=3.21	0.07
Albumin, g/L	0.97	0.91–1.03	χ²=1.38	0.24
Stage III vs II-high risk	1.32	0.75–2.32	χ²=0.98	0.32

Values are HR with 95% CI from multivariable Cox proportional hazards regression. *P<0.05 significant.

CEA >5 ng/mL (HR 1.52, 95% CI 0.95–2.46, P = 0.08) and LDH elevation (HR 1.69, 95% CI 0.94–3.06, P = 0.07) showed borderline associations with DFS, suggesting potential but inconclusive prognostic relevance, whereas albumin (HR 0.97, 95% CI 0.91–1.03, P = 0.24) and stage (HR 1.32, 95% CI 0.75–2.32, P = 0.32) were not significantly associated with DFS. These results are summarised in **Table 4**.

### 3.4 Temporal trajectories of serum IgG and CEA

**Figure 3:**
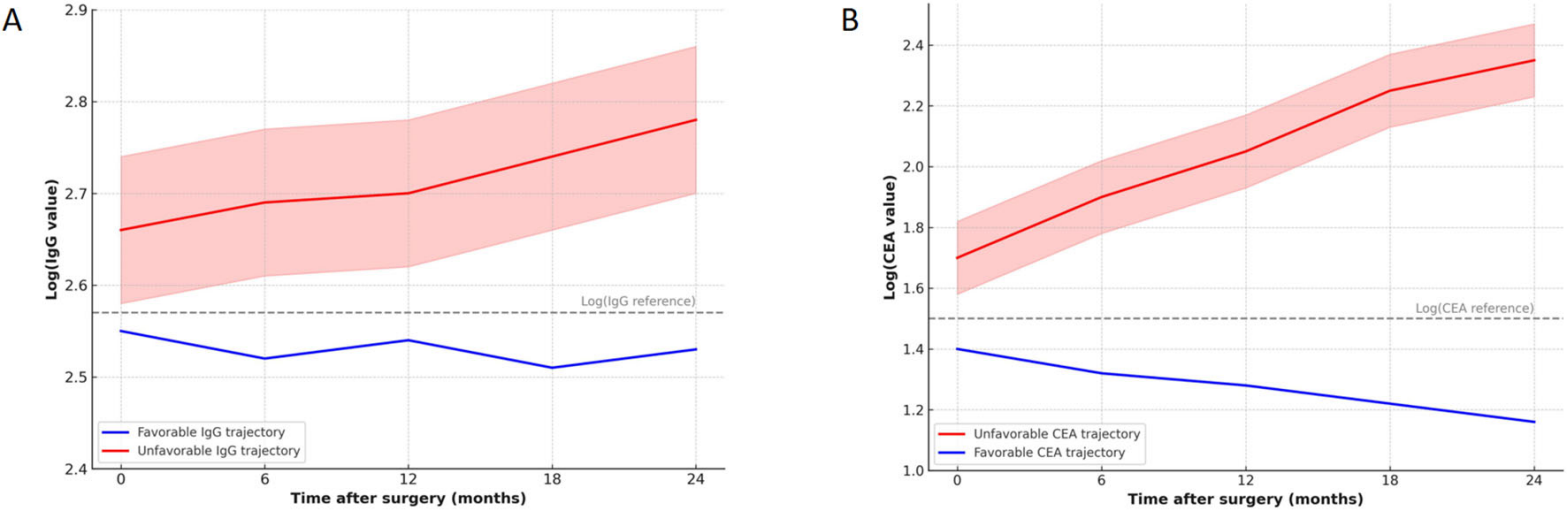
Temporal trajectories of serum IgG and CEA following curative resection for high-risk stage II–III CRC.

Serial assessments of serum immunoglobulin G (IgG) and carcinoembryonic antigen (CEA) up to 24 months after curative resection revealed distinct temporal patterns across patient subgroups (**Figure 3**). Patients with favourable IgG trajectories (n=110) demonstrated a modest decline from postoperative baseline values (mean log-transformed IgG 2.75 at baseline to 2.62 at 24 months), remaining close to reference thresholds with minor fluctuations. By contrast, unfavourable IgG trajectories (n=82) were characterised by a progressive increase over time, with mean log-transformed levels rising from 2.70 at baseline to 2.87 at 24 months. This divergence in temporal dynamics indicated clear biological separation between subgroups.

Parallel analyses of CEA yielded similar patterns, though with less pronounced separation. Favourable CEA trajectories (n=120) showed a steady decrease from a mean log-transformed value of 1.40 at baseline to 1.16 at 24 months, whereas unfavourable trajectories (n=72) rose from 1.70 to 2.35 over the same period. The unfavourable group also exhibited wider variability, as reflected in broader confidence intervals, suggesting greater heterogeneity in disease biology. Despite these upward shifts, the magnitude of separation between favourable and unfavourable CEA trajectories was less marked than that observed for IgG. Taken together, the temporal dynamics of IgG and CEA established two distinct monitoring profiles, which served as the foundation for subsequent survival analyses.

### 3.5 Dynamic monitoring of IgG and CEA

**Figure 4:**
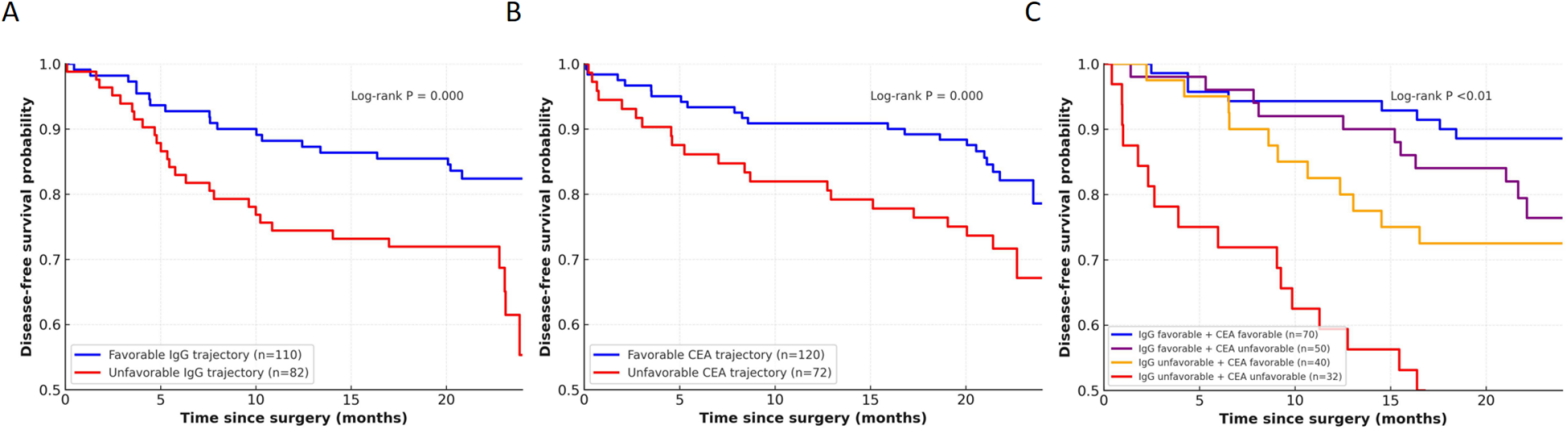
Kaplan–Meier survival curves according to dynamic monitoring of IgG and CEA. **(A)** IgG trajectory groups. **(B)** CEA trajectory groups. **(C)** Combined IgG/CEA trajectory groups. Number at risk is shown below the x-axis.

Dynamic changes in serum IgG were evaluated in 192 patients with serial measurements up to 24 months after surgery. Patients were stratified into favourable (n=110) and unfavourable (n=82) trajectories. As shown in **Figures 4A**, unfavourable IgG trajectories were strongly associated with inferior 2-year DFS compared with favourable trajectories (55% [95% CI 44–65] vs 82% [95% CI 73–88], log-rank P<0.01).

In contrast, dynamic monitoring of CEA showed only a borderline separation between favourable (n=120) and unfavourable (n=72) trajectories, with estimated 2-year DFS rates of 79% (95% CI 71–85) and 67% (95% CI 55–77), respectively (log-rank P = 0.06; **Figure 4B**). When combining IgG and CEA trajectories, four groups were defined. As shown in **Figures 4C**, a clear stepwise gradient was observed: patients with both favourable IgG and CEA trajectories achieved the best outcome (2-year DFS 89% [95% CI 79–94]), followed by those with IgG favourable/CEA unfavourable (76% [95% CI 62–86]) and IgG unfavourable/CEA favourable (73% [95% CI 56–84]), whereas patients with both unfavourable trajectories had the poorest survival (2-year DFS 31% [95% CI 16–47]). The overall difference among groups was significant (log-rank P<0.01).

### 3.6 Multivariable analysis of dynamic IgG and CEA trajectories

In the multivariable Cox proportional hazards regression model including dynamic IgG and CEA trajectories along with relevant clinicopathological covariates, unfavourable IgG trajectory emerged as an independent predictor of inferior 2-year DFS (HR 2.05, 95% CI 1.32–3.18; P = 0.002) (**Table 5**). By contrast, unfavourable CEA trajectory showed only a modest, non-significant trend towards worse outcomes (HR 1.36, 95% CI 0.88–2.10; P = 0.16).

**Table 5:** Multivariable Cox regression analysis of dynamic IgG and CEA trajectories.

Variable	HR	95% CI	Statistical test (Wald χ²)	P value
Dynamic IgG trajectory (unfavourable vs favourable)	2.05	1.32–3.18	χ²=9.39	0.002*
Dynamic CEA trajectory (unfavourable vs favourable)	1.36	0.88–2.10	χ²=1.96	0.16
KRAS mutation (yes vs no)	1.71	1.02–2.85	χ²=4.16	0.04*
Stage III vs II-high risk	1.28	0.74–2.21	χ²=0.80	0.37
Albumin (per g/L increase)	0.96	0.90–1.03	χ²=1.39	0.24

Values are HR with 95% CI from multivariable Cox proportional hazards regression. *P<0.05 significant.

Among other covariates, KRAS mutation was independently associated with decreased DFS (HR 1.71, 95% CI 1.02–2.85; P = 0.04), whereas disease stage (stage III vs high-risk stage II, HR 1.28, 95% CI 0.74–2.21; P = 0.37) and baseline albumin level (per g/L increase, HR 0.96, 95% CI 0.90–1.03; P = 0.24) were not significantly associated with prognosis. These results are summarised in **Table 5**.

## 4 Discussion

In this retrospective cohort study of patients with high-risk stage II and stage III colorectal cancer, we observed that dynamic monitoring of serum IgG provided significant prognostic information beyond conventional clinicopathological features and established serological markers. Patients with unfavourable IgG trajectories had markedly worse two-year disease-free survival compared with those with favourable trajectories, and this association remained robust in multivariable models adjusting for KRAS mutation, CEA, albumin, and disease stage. In contrast, dynamic CEA monitoring, although widely adopted in clinical practice, demonstrated only borderline discrimination, and its effect was not statistically significant after adjustment. These findings suggest that serial IgG assessment may complement traditional prognostic tools in refining postoperative risk stratification.

Previous studies have largely focused on CEA and pathological risk features as the mainstay for surveillance in colorectal cancer (16). While elevated preoperative CEA has long been associated with adverse prognosis, its limited sensitivity and specificity have been well documented (17, 18). Our study corroborates this limitation by showing that CEA dynamics were not independently predictive, whereas IgG, a non-traditional immune-related biomarker, emerged as a significant determinant of recurrence risk. Although evidence linking immunoglobulin levels to cancer outcomes is relatively sparse, earlier reports have suggested associations between immunoglobulin abnormalities and immune dysregulation in gastrointestinal malignancies (19). Our results expand on these observations by demonstrating the prognostic utility of IgG dynamics in a well-defined surgical cohort.

The biological rationale for this finding may lie in the interaction between systemic immunity and tumour progression (20, 21). IgG, as the dominant circulating immunoglobulin, reflects the activity of B-cell mediated responses and may also serve as a surrogate for systemic inflammatory states. Persistent or rising IgG levels after surgery could indicate ongoing immune activation or subclinical disease persistence, thereby identifying individuals at higher risk of recurrence (11, 22). This hypothesis is consistent with prior work highlighting the prognostic role of systemic immune-inflammation indices, although our analysis provides a more specific focus on humoral immunity (23–25). Importantly, by incorporating longitudinal measurements rather than a single baseline value, we captured temporal changes in immune status that may better mirror disease dynamics.

Clinically, these findings suggest that IgG measurement may have two complementary contexts: baseline preoperative risk stratification (**Figure 1**) and postoperative longitudinal surveillance (**Figure 4**) (26, 27). Although our study is prognostic and does not test interventions, an unfavourable postoperative IgG trajectory could be used to support risk-adapted management actions in routine practice, such as (i) repeat testing with evaluation for intercurrent infection/inflammation or other non-malignant causes of IgG elevation, (ii) shortening the interval of follow-up visits/serum marker monitoring, and (iii) considering earlier cross-sectional imaging or other recurrence work-up within guideline-concordant surveillance pathways. Moreover, the combined analysis of IgG and CEA trajectories revealed a stepwise gradient of risk, supporting the concept that integrating immune-related biomarkers with conventional markers may yield a more nuanced prognostic model (28, 29). Whether IgG-guided surveillance or treatment intensification can improve outcomes will require prospective interventional validation (30, 31).

Several limitations must be acknowledged. First, the retrospective single-centre design may limit generalizability, and residual confounding cannot be excluded despite adjustment for key covariates. Second, patients who received neoadjuvant (preoperative) chemotherapy and/or radiotherapy were not included; therefore, the applicability of these findings to rectal cancer populations treated with neoadjuvant chemoradiotherapy remains uncertain. Third, the trajectory definitions relied on prespecified percentage-change thresholds (e.g., 15% for IgG) without formal sensitivity analyses across alternative cut-offs; thus, the optimal threshold and clinical decision limits require external validation. Fourth, because biomarker trajectories are derived from serial measurements accrued during follow-up, modelling them as fixed covariates may introduce guarantee-time (immortal-time) bias; future work should apply time-dependent covariate models or landmark analyses to strengthen causal interpretation. Finally, the follow-up period was restricted to two years, which may underestimate late recurrences, and we did not incorporate mechanistic studies to directly link IgG changes with immune microenvironment alterations. These factors underscore the need for prospective, multicentre studies with longer follow-up and external validation to confirm our findings and clarify the underlying biology.

## 5 Conclusion

In this retrospective cohort of patients with high-risk stage II and stage III colorectal cancer, dynamic monitoring of serum IgG was independently associated with postoperative disease recurrence and provided prognostic value beyond conventional factors including CEA and stage. These findings suggest that serial IgG assessment may serve as a complementary biomarker to refine risk stratification, although confirmation in prospective multicentre studies with longer follow-up is warranted.

## Data Availability

The raw data supporting the conclusions of this article will be made available by the authors, without undue reservation.
